# Lymph node status as a prognostic factor after palliative resection of primary tumor for patients with metastatic colorectal cancer

**DOI:** 10.18632/oncotarget.15696

**Published:** 2017-02-25

**Authors:** Qingguo Li, Changjian Wang, Yaqi Li, Xinxiang Li, Ye Xu, Guoxiang Cai, Peng Lian, Sanjun Cai

**Affiliations:** ^1^ Department of Colorectal Surgery, Fudan University Shanghai Cancer Center, Shanghai, China; ^2^ Department of Oncology, Shanghai Medical College, Fudan University, Shanghai, China; ^3^ Anorectal Department, Hangzhou Third Hospital, Hangzhou, China

**Keywords:** metastatic colorectal cancer, palliative resection, lymph node status, survival analysis

## Abstract

Lymph node (LN) status is one of the most important predictors for M0 colorectal cancer patients. However, its clinical impact on stage IV colorectal cancer remains unclear. The study aimed to explore the prognostic value of LN status after palliative resection of primary tumor for patients with metastatic colorectal cancer (mCRC). We combined analyses of mCRC patients in Surveillance, Epidemiology and End Results (SEER) database and Fudan University Shanghai Cancer Center (FUSCC). A total of 17,553 patients with mCRC were identified in SEER database. X-tile program was adopted to identify 2 and 10 as optimal cutoff values for negative lymph node (NLN) count to divide patients into 3 subgroups of high, middle and low risk of cancer related death. N stage and NLN count were verified as independent prognostic factors in multivariate analyses of patients in whole cohort and in subgroup analyses of each N stage (*P*<0.05). Validation of FUSCC cohort of patients demonstrated that metastatic tumor burden (*P* = 0.042), NLN count (*P* = 0.039) and sequential chemotherapy (*P* = 0.040) were significant predictors of poorer CSS. Specifically, the prognosis of patients at stage N0 was significantly more favorable than that of patients at stage N2 (*P* = 0.038). In conclusion, primary tumor LN status was a strong predictor of CSS after palliative resection of metastatic colorectal cancer. Advanced N stage and small number of NLN were correlated with high risk of cancer related death after palliative resection of primary tumor.

## INTRODUCTION

Colorectal cancer (CRC) is a leading cause of cancer mortality in the world [1]. The prognosis of CRC patients is mainly determined by clinical staging system at the time of diagnosis. Approximately 20-25% of patients present with metastatic disease (stage IV) [2], and the incidence of stage IV cancer seems increasing [3]. Generally, resection of primary tumor is not recommended unless R0 resection is feasible or patients are symptomatic or anticipated to experience potential tumor complications, such as obstruction, perforation or bleeding [4].

However, whether palliative resection of the primary tumor actually offers a survival benefit for patients with metastatic colorectal cancer (mCRC) is controversial [5–11]. A number of retrospective studies have suggested that primary tumor resection is safe and associated with improved outcomes in mCRC patients [5–8]. Meta-analyses of retrospective studies suggest that mCRC patients undergoing primary tumor resection combined with systemic therapy have more favorable survival compared with patients treated with systemic therapy alone [12–15]. Factors associated with better survival include secondary curative surgery, well-differentiated primary tumor, exclusive liver metastases, and sequential chemotherapy [8, 16].

Lymph node (LN) status is one of the most important predictors for CRC patients without metastasis [17–20]. However, its clinical impact on mCRC patients remains unclear. With advances in medical technologies, surgical therapies for mCRC have been verified linked to survival benefits. Therefore, it is important to explore whether the prognostic value of LN status in primary tumor is clinically significant in patients with mCRC. Our study aimed at assessing impact of the LN status on survival of mCRC patients after palliative surgery of primary tumor adopting SEER database and validation cohort of patients from Fudan University Shanghai Cancer Center (FUSCC).

## RESULTS

### Patient characteristics in SEER database

A total of 17,553 patients with mCRC were identified in SEER database. The median age of the cohort was 63 years (IQR, 53-73 years) with a majority of patients being White in race (*n* = 13,464, 76.7%). The primary site of over four-fifths of patients’ was colon (*n* = 14,754, 84.1%), while 15.9% (*n* = 2,799) of the cohort was rectum. The median LN retrieval was 14.0 (IQR, 10.0-20.0), with approximately half of all patients presenting with N2 stage (*n* = 9,214, 52.5%), 30.4% (*n* = 5,334) of patients with N1 stage and 17.1% (*n* = 3,005) with N0 stage. Patients’ demographics and pathological features are summarized in Table 1.

**Table 1:** Clinical characteristics of patients with metastatic colorectal cancer treated with palliative surgery of primary tumor in SEER and FUSCC cohort

Variable		SEER Cohort		FUSCC Cohort	
		*N*	%	*N*	%
Sex					
	Male	9071	51.7	239	61.0
	Female	8482	48.3	153	39.0
Age		63	53-73	58	50-65
Race					
	White	13464	76.7	/	/
	Black	2528	14.4	/	/
	Others*	1561	8.9	/	/
Primary site					
	Colon	14754	84.1	230	58.7
	Rectum	2799	15.9	162	41.3
Grade					
	High/ Moderate	11520	65.6	254	64.8
	Poor/ Anaplastic	5509	31.4	138	35.2
	Unknown	524	3.0	/	/
T stage					
	T1-2	611	3.5	17	4.3
	T3	10621	60.5	53	13.5
	T4	6177	35.2	322	82.1
	Tx	144	0.8	/	/
N stage					
	N0	3005	17.1	98	25.0
	N1	5334	30.4	129	32.9
	N2	9214	52.5	165	42.1
No. of LNs					
	<12	5747	32.7	96	24.5
	≥12	11806	67.3	296	75.5
No. of NLNs					
	0-2	2876	16.4	36	9.2
	3-10	7196	41.0	161	41.1
	≥11	7486	42.6	195	49.7
M stage					
	One organ	/	/	325	82.9
	>1 organs	/	/	67	17.1

**Figure 1:**
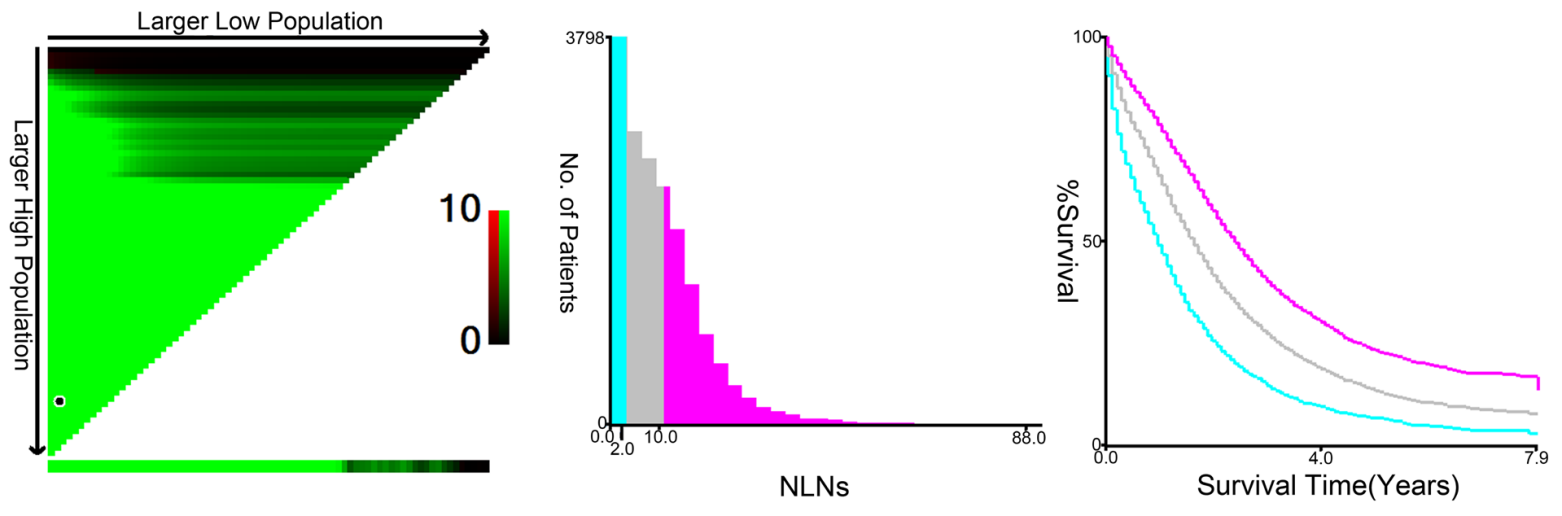
X-tile analysis of survival data from the SEER registry X-tile analysis was done on patients from the SEER database, who were equally divided into training and validation sets. X-tile plots of training sets are shown in the left panels, with plots of matched validation sets shown in the smaller inset. The optimal cut-point highlighted by the black circle in the left panels is shown on a histogram of the entire cohort (middle panels) and a Kaplan-Meier plot (right panels). *P* values were determined by using the cut-point defined in the training set and applying it to the validation set. Figures show LNR divided at the optimal cut-point (2 and 10, χ2 = 1209.449, *P < 0.001*).

The median number of NLNs for patients included in SEER database was 9 (IQR, 4-15). X-tile plots were constructed and the maximum of χ^2^ log-rank value of 1209.449 was produced applying the number 2 and 10 as cutoff values to divide the cohort into high, middle, and low subsets in terms of CSS (Figure 1).

### Impact of N stage, total lymph node (TLN) count, and NLNs on survival of patients in SEER database

Median follow-up time for the entire cohort was 17 months. At the end of the follow-up time, 12,424(70.8%) patients died of CRC, and 1-, 3-, and 5-year CSS were 67.0%, 30.0%, and 16.0%, respectively. LN status differences were noted in CSS.

**Figure 2:**
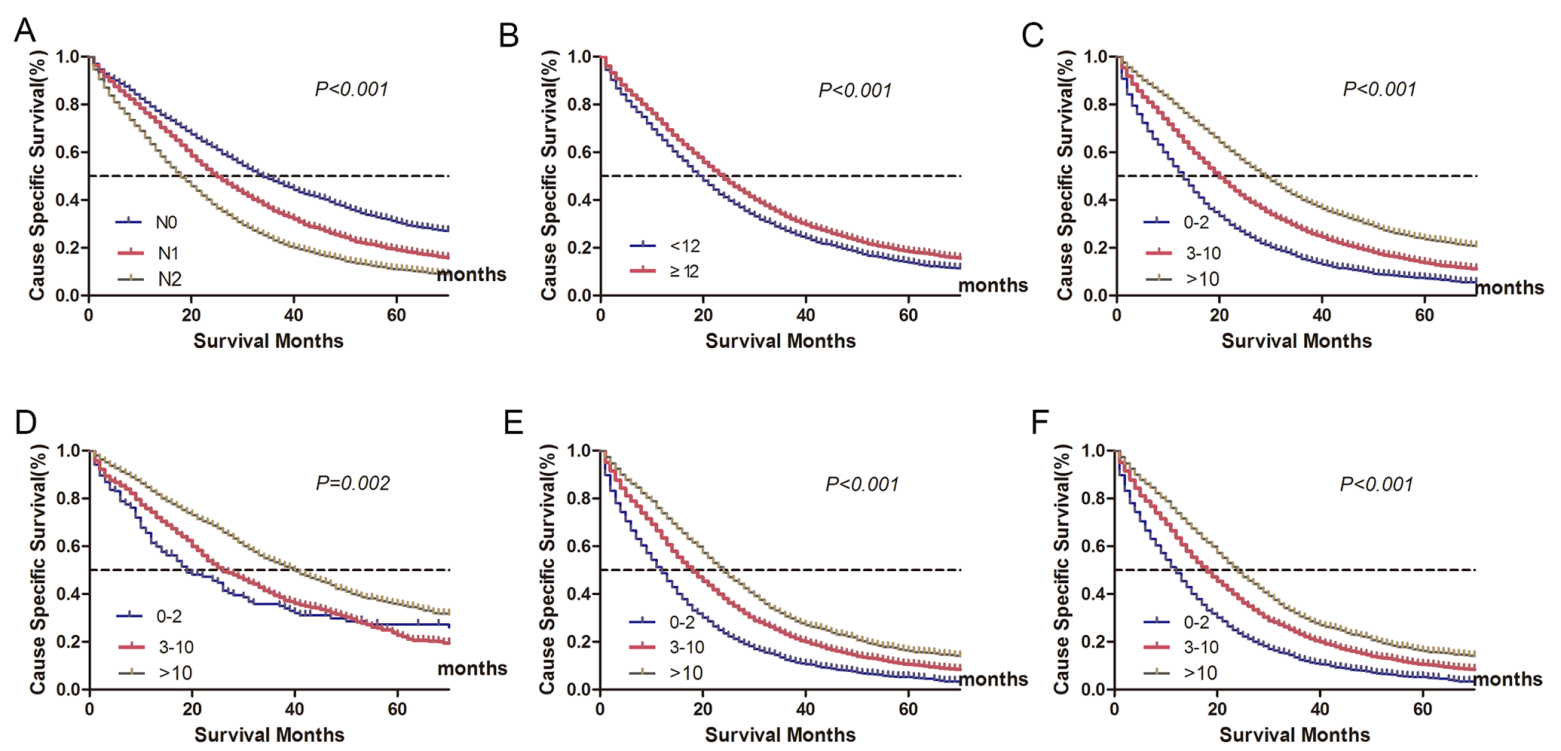
Cancer-specific survival (CSS) stratified by different lymph node status (A-C) **A**. N stage: 5-year CSS were 29.6%, 18.4%, 10.4% in N0, N1 and N2 stage, respectively (χ2 = 727.324, *P < 0.001*). **B**. Lymph node count: 5-year CSS were 13.0%, and 17.8% in patients with < 12 or ≥12 lymph node retrieval (χ2 = 134.373, *P < 0.001*). **C**. Negative lymph node count: 5-year CSS were 6.6%, 13.1%, 23.0% in patients with 0-2, 3-10 and ≥11 negative lymph node count, respectively (χ2 = 1209.449, *P < 0.001*). Log-rank tests of cancer-specific survival comparing those who had 0-2, 3-10 and ≥11 negative lymph node count(D-F). **D**. N0 stage: 25.3% *vs* 21.5% *vs* 34.8%, respectively; χ2 = 88.333, *P < 0.001*; **E**. N1 stage: 8.8% *vs* 15.2% *vs* 22.8%, respectively; χ2 = 192.418, *P < 0.001*; **F**. N2 stage: 4.8% *vs* 10.0% *vs* 15.7%, respectively; χ2 = 531.534, *P < 0.001*.

The survival impact of N stage, TLN, and NLN are shown in Table 2 and Figure 2A-2C. Concerning N stage, we found that mCRC patients with N0 stage had significantly better prognosis than non-N0 patients in univariate analysis (χ^2^ = 727.324, *P* < 0.001). Patients who had < 12 LNs retrieval at the time of pathological evaluation had a higher risk of cancer-specific mortality than those with ≥ 12 LN retrieval, with 5-year CSS of 13.0% ( < 12 LNs retrieval) and 17.8% (≥12 LNs retrieval), respectively. These differences in survival were also noted after the cohort was stratified by NLN. Specifically, 5-year CSS was the highest among patients with ≥11 NLNs count: 23.0% *vs*. 3-10 NLN count: 13.1% *vs*. 0-2 NLN count: 6.6 %( χ2 = 1209.449, *P* < 0.001).

**Table 2:** Univariate and multivariate analyses for evaluating the influence of the lymph node status on CSS for mCRC patients in SEER database

		Univariate analysis		Multivariate analysis	
Variable	5-year CSS	Log rank χ^2^ test	*P*	HR (95%CI)	*P*
Primary site		86.766	<0.001		<0.001
Colon	15.7%			Reference	
Rectum	18.4%			0.849(0.808-0.892)	
Sex		3.916	0.048		0.412
Male	15.7%			Reference	
Female	16.6%			1.015(0.980-1.051)	
Age		460.646	<0.001		<0.001
<60	20.0%			Reference	
≥60	13.0%			1.505(1.451-1.560)	
Race		38.942	<0.001		<0.001
White	16.6%			Reference	
Black	12.1%			1.171(1.114-1.231)	<0.001
Others*	18.6%			0.858(0.803-0.916)	<0.001
Grade		635.909	<0.001		0.125
High/ Moderate	18.4%			Reference	
Poor/ Anaplastic	11.0%			1.418(1.364-1.475)	<0.001
Unknown	19.7%			1.083(0.971-1.208)	0.154
Histotype		80.662	<0.001		0.125
Adenocarcinoma	16.1%			Reference	
Mucinous Adenocarcinoma	17.8%			1.007 (0.953-1.065)	0.801
signet ring cell	6.6%			1.120(1.004-1.250)	0.042
T Stage		314.863	<0.001		<0.001
T1-2	28.4%			Reference	
T3	17.5%			1.188(1.069-1.321)	0.001
T4	12.0%			1.450(1.301-1.615)	<0.001
Tx	31.6%			0.971(0.763-1.235)	0.809
N stage		727.324	<0.001		<0.001
N0	29.60%			Reference	
N1	18.40%			1.301(1.228-1.379)	<0.001
N2	10.4%			1.605(1.512-1.701)	<0.001
No. of LNs		134.373	<0.001		0.609
<12	13.0%			Reference	
≥12	17.8%			1.013(0.964-1.065)	
No. of NLNs		1209.449	<0.001		<0.001
0-2	6.6%			Reference	
3-10	13.1%			0.701(0.667-0.737)	<0.001
≥11	23.0%			0.516(0.483-0.551)	<0.001

NI: not included in the multivariate survival analysis.

Others including American Indian/Alaska native, Asian/Pacific Islander, and unknown

In the multivariable Cox regression model adjusted for primary site, sex, age, race, tumor grade, histotype, T stage, N stage, TLN count and NLN count, the association among N stage and NLN classification with CSS persisted, but TLN count lost its significance (*P* = 0.609). The adjusted hazard ratios (HRs) and 95 % confidence intervals (CIs) for N1 and N2 were 1.301 (1.228-1.379, *P* < 0.001) and 1.605 (1.512-1.701, *P* < 0.001), respectively. For NLN classification, a higher number of NLNs was found to have a protective effect on survival (3-10 counts, HR 0.701, 95%CI 0.667-0.737; ≥11 counts, HR 0.516, 95%CI 0.483-0.551, 0-2 was used as reference) (Table 3).

As N stage was a well-accepted prognostic predictor for stage-III CRC, we then made further analysis to explore whether NLN count was a prognostic predictor for mCRC patients in each N stage. The results showed that the protective value of high NLN counts persisted in each N stage (*P* < 0.001) (Table 3, Figure 2D-2F).

**Table 3:** Univariate and multivariate analyses of NLN count on CSS based on different N stages for mCRC patients in SEER database

		Univariate analysis		Multivariate analysis	
Variable	5-year CSS	Log rank χ^2^ test	*P*	HR (95%CI)	*P*
N Stage					
N0 stage					
No. of NLNs		88.333	<0.001		0.002
0-2	25.3%			Reference	
3-10	21.5%			0.865(0.706-1.059)	0.161
≥11	34.8%			0.596(0.443-0.802)	0.001
N1 stage					
No. of NLNs		192.418	<0.001		<0.001
0-2	8.8%			Reference	
3-10	15.2%			0.734(0.657-0.819)	<0.001
≥11	22.8%			0.612(0.506-0.739)	<0.001
N2 stage					
No. of NLNs		531.534	<0.001		<0.001
0-2	4.8%			Reference	
3-10	10.0%			0.664(0.624-0.705)	<0.001
≥11	15.7%			0.503(0.467-0.542)	<0.001

*P*-values refer to comparisons between the groups and were adjusted for primary site, sex, age, race, pathological grading, tumor histotype, the number of LNs retrieval, and N stage as covariates.

### Evaluating the SEER database outcomes in FUSCC cohort

Of 392 eligible patients identified in FUSCC, no patients received secondary curative surgery for metastases in follow-up time. 325 (82.9%) patients have distant metastases confined to one organ. 46 (11.7%) patients received chemotherapy for less than 3 cycles.

**Figure 3:**
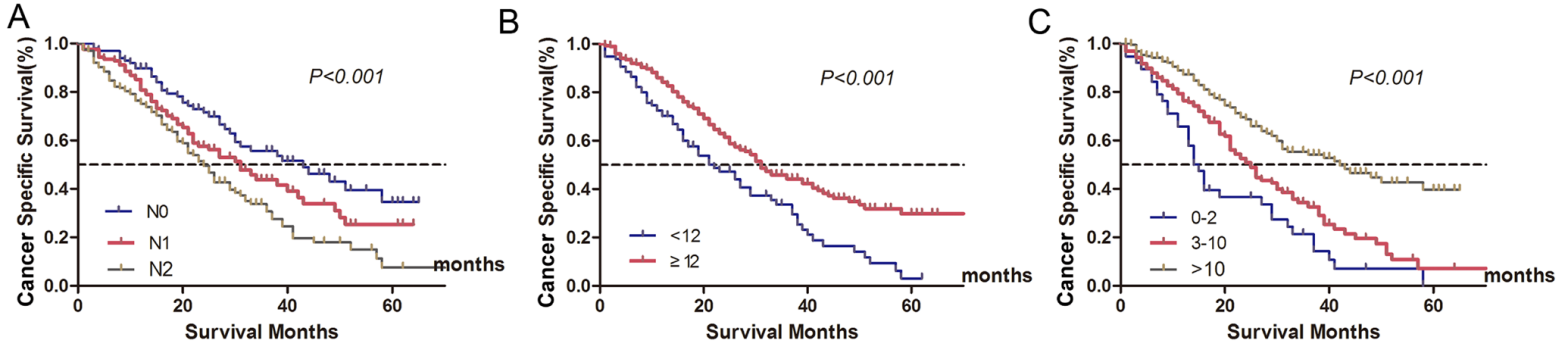
Survival curves of metastatic colorectal cancer treated with palliative surgery in Fudan University Shanghai Cancer Center according to different lymph node status **A**. N stage: 5-year CSS were 34.3%, 24.3%, 7.6% in N0, N1 and N2 stage, respectively (χ2 = 17.492, *P < 0.001*). **B**. Lymph node count: 5-year CSS were 2.9%, and 29.4% in patients with < 12 or ≥12 lymph node retrieval (χ2 = 15.047, *P < 0.001*). **C**. Negative lymph node count: 5-year CSS were 0, 6.6%, 39.1% in patients with 0-2, 3-10 and ≥11 negative lymph node count, respectively (χ2 = 39.215, *P < 0.001*).

After the median follow-up time of 19 months, 201(51.3%) patients died of CRC. In univariate analysis, factors associated with CSS were tumor grade, N stage, metastatic tumor burden, CEA level, number of LN retrieval, NLN count, and sequential chemotherapy (Table 4, Figure 3). In multivariate analysis, metastatic tumor burden (*P* = 0.042), NLN count (*P* = 0.039), and sequential chemotherapy (*P* = 0.040) were significant predictors of poorer CSS (Table 4). Specifically, the prognosis of N0 patients was significantly more favorable than that of N2 patients (*P* = 0.038), although there was no significant difference between N0 and N1 patients (*P* = 0. 112).

**Table 4:** Univariate and multivariate analyses for evaluating the influence of the lymph node status on CSS for mCRC patients in FUSCC database

		Univariate analysis		Multivariate analysis	
Variable	5-year CSS	Log rank χ^2^ test	*P*	HR(95%CI)	*P*
Primary site		0.115	0.734		NI
Colon	17.4%				
Rectum	22.5%				
Sex		1.643	0.200		NI
Male	24.9%				
Female	17.0%				
Age		0.001	0.969		NI
<60	21.8%				
≥60	19.4%				
Grade		6.334	0.012		0.496
High/ Moderate	25.9%			Reference	
Poor/ Anaplastic	13.0%			1.113(0.818-1.513)	
Histotype		0.048	0.976		NI
Adenocarcinoma	21.2%				
Mucinous Adenocarcinoma	22.0%				
signet ring cell	50.0%				
T Stage		2.333	0.311		NI
T1-2	16.6%				
T3	34.8%				
T4	17.1%				
N stage		17.492	<0.001		0.109
N0	34.3%			Reference	
N1	24.3%			1.393(0.926-2.092)	0.112
N2	7.6%			1.585(1.026-2.448)	0.038
Metastatic Tumor burden		10.039	0.002		0.042
One organ	24.8%			Reference	
Multiple organs	0			1.422(1.013-1.995)	
CEA		8.415	0.004		0.200
Normal	24.1%			Reference	
High	16.8%			0.827(0.618-1.106)	
No. of LNs		15.047	<0.001		0.076
<12	2.9%			Reference	
≥12	29.4%			0.721(0.502-1.035)	
No. of NLNs		39.215	<0.001		0.039
0-2	0			Reference	
3-10	6.6%			0.719(0.478-1.082)	0.114
≥11	39.1%			0.505(0.298-0.855)	0.011
Sequential chemotherapy		8.386	0.004		0.040
<3 cycles	0			Reference	
≥3 cycles	23.1%			0.664(0.449-0.982)	

NI: not included in the multivariate survival analysis.

## DISCUSSION

This is the first study to systematically evaluate the clinical implications of LN status in mCRC patients based on data from two independent databases. Our study showed that LN metastasis status and NLN count are crucial for predicting survival outcome after palliative resection of primary tumor in mCRC. We first got the conclusion from SEER database, served as a reflection of general practice rather than the practice of tertiary care centers alone, and then validated the findings in FUSCC cohort.

In recent years, many studies have reported a survival benefit for mCRC patients undergoing palliative primary tumor resection [5–8, 12–14]. However, the prognostic impact of LN status remains unclear. Although two studies indicated that LN ratio (LNR) was associated with survival outcomes after palliative resection of mCRC [21, 22]. However, the sample was small. Also, the LNR was calculated from positive LN (PLNs) counts and TLN counts. TLN counts comprises both PLNs and NLNs, so the relationship between LNR or TLNs and prognosis is confounded by the prognostic effect of the number of PLNs. TLN count was validated with no apparent relationship with CSS both in SEER and FUSCC cohorts.

The prognostic impact of N stage, especially the protective effect of NLN count on patients with mCRC observed in the present study is interesting, because removal of regional LNs theoretically does not improve the survival of mCRC patient with apparently more serious metastatic lesions than primary tumor. Also, N stage and the number of LN retrieval is not included in the stratification of stage IV CRC by the current 7^th^ edition UICC/AJCC TNM grading system. More LNs dissected may cause expanded tissue damage and may increase patients’ comorbidity and mortality rates in stage IV CRC. However, in order to explore the potential impact of NLN count on the prognostic prediction of mCRC patients, we further analyzed the impact of NLN count in each N stage subgroup and found that the value of NLN counts as a prognostic factor still persisted, which suggested that protective effect of NLN count existed irrespective of N stage, and dissection of non-metastatic LNs improved the prognosis of mCRC patients without regional LN metastasis.

Some previously published articles support our findings. Ishihara S *et al* reported that D3 LN dissection, which is also described as high tie or central vascular ligation, consisting of removal of LNs up to the origin of the feeding artery, was significantly associated with a better OS of patients with mCRC [23]. In metastatic gastric cancer, the number of dissected nodes were also significantly associated with postoperative survival benefits [24].

Although the survival benefit of more NLN retrieval is observed, the underline mechanism is still unknown. One hypothesis relates to cancer immunity, indicating that the immunity exerted by tumor-draining LNs has dual function on cancer cells, namely antitumor immunity and tolerance for cancer, and that the balance of cancer immunity inclines to immune tolerance as the cancer advances [25]. Resection of regional LNs might reset the immunological balance, resulting in an improvement of patients’ prognosis. Another hypothesis is that surgical resection is a work of art. High percentage of patients with mCRC have tumor cell infiltrated through bowel wall, which can be illustrated by the fact that 95.7% in SEER database and 95.6% in FUSCC database were staged as T3 and T4. High number of NLN retrieval may reflect of delicate surgical resection. Improved surgical techniques may reduce the chances of iatrogenic spread of cancer cells [18].

The present study has potential limitations. First, as publicly available database, the SEER registry does not collect data on chemotherapy, metastatic tumor burden, and secondary curative surgery. However, the large sample size is necessary for the study and we addressed relevant issues in FUSCC cohort of patients. Second, preoperative comorbidity and mortality were main concerns for palliative resection of mCRC patients. As we mainly focused on prognostic factors associated with long-term outcomes, these factors were not included in the study. Third, the number of patients in FUSCC cohort was relatively small. Factors, such as age and T stage, which were validated as independent prognostic factors in SEER database, were not confirmed in FUSCC cohorts. But since we mainly focused on LN status and survival outcomes in mCRC patients, we believed this disparity doesn't impair the power of our study.

Our study revealed that primary tumor LN status was a strong predictor of CSS after palliative resection of mCRC. Advanced N stage and small number of NLN were correlated with high risk of cancer related death after palliative resection of primary tumor. Standard LN dissection may be still necessary for palliative resection of primary tumor for mCRC patients.

## PATIENTS AND METHODS

### Patient selection in the SEER database

To minimize variation in systemic treatment regimens, only patients submitted in November 2015 to SEER database were identified. Inclusion criteria included: (1) patients were diagnosed from 2004 to 2010; (2) the site code represented “colon” (C18.0–C18.9); “rectosigmoid junction” (C19.9), and “rectum” (C20.9) according to *Third Edition of International Classification of Diseases for Oncolog*y (ICD-O-3); (3) histology codes denoted adenocarcinoma (8150/3, 8210/3, 8261/3, 8263/3), mucinous adenocarcinoma (8480/3), or signet ring cell carcinoma (8490/3); (4) patients were with distant metastasis(M1); (5) patients had undergone primary tumor resection; (6) patients were with 1 or more regional nodes examined; (7) patients were without radiotherapy before surgery; (8) age of patients was limited to between 18 and 80 years old; (9) CRC was the only type of primary; (10) information on cancer-specific survival (CSS) and survival months were available.

We collected the following data: age at diagnosis, sex, race, year of diagnosis, primary site labeled, radiation sequence with surgery, reason no cancer directed surgery, tumor size, tumor histology, number of primaries, sequence number, and information on CSS. All data were obtained from the SEER-stat software (SEER*Stat 8.1.5) of the National Cancer Institute in the United States. All cases were restaged according to the American Joint Committee on Cancer (AJCC) Cancer Staging Manual (7th edition, 2010). Patient race was categorized as white, black, and others (including American Indian/Alaska native, Asian/Pacific Islander, and unknown) based on SEER coding scheme. For ease of analysis, tumor grade was categorized as a binary variable combing grade I and II into a single category and grade III and IV into another. CSS was measured from the date of diagnosis to the date of death from CRC.

### Patient selection in the FUSCC cohort

The FUSCC CRC dataset was built prospectively and recorded information of CRC patients treated at FUSCC, Shanghai, China since January 2006 [18, 19]. To validate the findings from the SEER database and to clarify relevant issues, mCRC patients from the FUSCC treated with palliative resection of primary tumor between January 2006 and December 2010 were identified.

The inclusion criteria were carried out as mentioned above. All patients did not receive secondary curative surgery until death or the last follow-up on January 1, 2016. Distant metastases were dichotomized as restricted to one organ or multiple organs. The regimens used varied because of long duration of data collection. So, we simply classify patients into two groups according to whether patients had received more than three cycles of chemotherapy after surgery or not. The survival data was provided by clinical statistics center of FUSCC, relying on the hospital medical records follow-up platform or contacts with patients by phone or email. Patients who were alive at last follow-up were censored for analysis. All patients in FUSCC provided written informed consent. The Institutional Review Board of Fudan University Shanghai Cancer Center approved this study.

### Statistical analysis

Descriptive statistics was reported as medians with interquartile range (IQR) for continuous variables and as whole numbers and percentages for categorical variables. Cutoff values of negative lymph nodes (NLNs) were determined and analyzed using X-tile program (http://www.tissuearray.org/rimmlab/), which identified the cutoff with the minimum *P* values from log-rank χ^2^ statistics in terms of survival [26]. The Chi-square (χ^2^) test was used to compare patient baseline characteristics. Survival rate was generated using Kaplan-Meier curves, and the differences were compared with the log-rank test. A Cox proportional hazards regression model was then built to evaluate the risks of variables on CSS in CRC patients. Statistical evaluation was conducted with SPSS 22.0 (SPSS Inc., Chicago, IL, USA). All confidence intervals (CIs) were stated at the 95% confidence level. Statistical significance was defined as *P* < 0.05 (two-sided).
